# GATA2 deficiency in an adult with alveolar proteinosis, infections, lymphadenopathy with granulomatosis, and immune deficiency: case report

**DOI:** 10.3389/fimmu.2025.1608392

**Published:** 2025-11-13

**Authors:** Juliana Gabzdilová, Štefan Raffáč, Gabriela Krasňanská, Michal Konečný

**Affiliations:** 1 Department of Haematology and Oncohaematology, P.J.Šafárik Medical Faculty and L.Pasteur University Hospital, Kosice, Slovakia; 2 Department of Clinical Immunology and Allergology, L.Pasteur University Hospital and OKIA s.r.o, Košice, Slovakia; 3 Laboratory of Genomic Medicine, GHC GENETICS SK, Bratislava, Slovakia; 4 Faculty of Natural Sciences, Department of Biotechnology a Biology, University of Ss. Cyril and Methodius in Trnava, Trnava, Slovakia

**Keywords:** monocytopenia, immunodeficiency, infections, alveolar proteinosis, granulomatosis of lymphatic tissue, autoimmunity

## Abstract

GATA2 protein is an important hematopoietic transcription factor. GATA2 deficiency, caused by heterozygous mutation of GATA2, includes a group of diseases presented with monocytopenia, cellular immunodeficiency with marked susceptibility to infection (mycobacterial, fungal, and viral), myelodysplasia (MDS), leukemic transformation, vessel abnormalities, and deafness. Most data are available in case reports. The challenge is to increase the awareness about GATA2 deficiency and to find the best treatment protocol for the patient. Allogeneic hematopoietic stem cell transplantation (allo-HSCT) is the only curative treatment available. However, it is also associated with significant treatment-related morbidity and mortality. Therefore, determining the appropriate timing of allo-HSCT is critical. We present the case of a young adult man with severe lung alveolar proteinosis, granulomatous disease, infections, and autoimmune complications present before we confirm GATA2 as a newly described mutation. The main sign that led to the suspicion of GATA2 deficiency was prolonged monocytopenia and absence of monocyte in peripheral blood and bone marrow along with recurrent infections and alveolar proteinosis. The exact and early diagnosis of this disease is crucial. The late stage of the disease is associated with increased mortality and severe complications that contraindicate bone marrow transplantation or worsen survival. The clinical implication of this article is to improve awareness about the different clinical forms of GATA2 deficiency and the new mutations described in this field. Our aim is to share real-life experiences with disease development and treatment during the lifetime of a young patient.

## Introduction

GATA2 is a hematopoietic stem cell transcription factor. The first experiences with GATA2 deficiency were published as monocytopenia with susceptibility to non-tuberculosis mycobacterial infections, other atypical infections, and myelodysplasia.

MonoMAC syndrome was described as the first clinical manifestation of a GATA2 gene mutation. Over time, the understanding of GATA2 deficiency has expanded, and a broader spectrum of related disorders was revealed. These conditions highlight that GATA2 deficiency encompasses a range of clinical features extending beyond the original description of MonoMAC syndrome. GATA2 deficiency caused by a heterozygous mutation in GATA2 gene presents a vast spectrum of clinical phenotypes. This includes a group of diseases presented with increased susceptibility to infections, cytopenia, especially profound monocytopenia, changes in bone marrow that vary from mild hypocellularity to multilineage dysplasia, acute myeloid leukaemia, and fibrosis, vessel abnormalities, and deafness.

Most data are available in case reports. Profound monocytopenia should prompt a consideration of GATA2 deficiency. The challenge is to increase the awareness about GATA2 deficiency and to find the best treatment protocol for the patient. Allogeneic hematopoietic stem cell transplantation (allo-HSCT) is the only curative treatment available. However, it is also associated with significant treatment-related morbidity and mortality. Therefore, determining the appropriate timing of allo-HSCT is critical (1, 2).

## Case report

**Figure 1:**
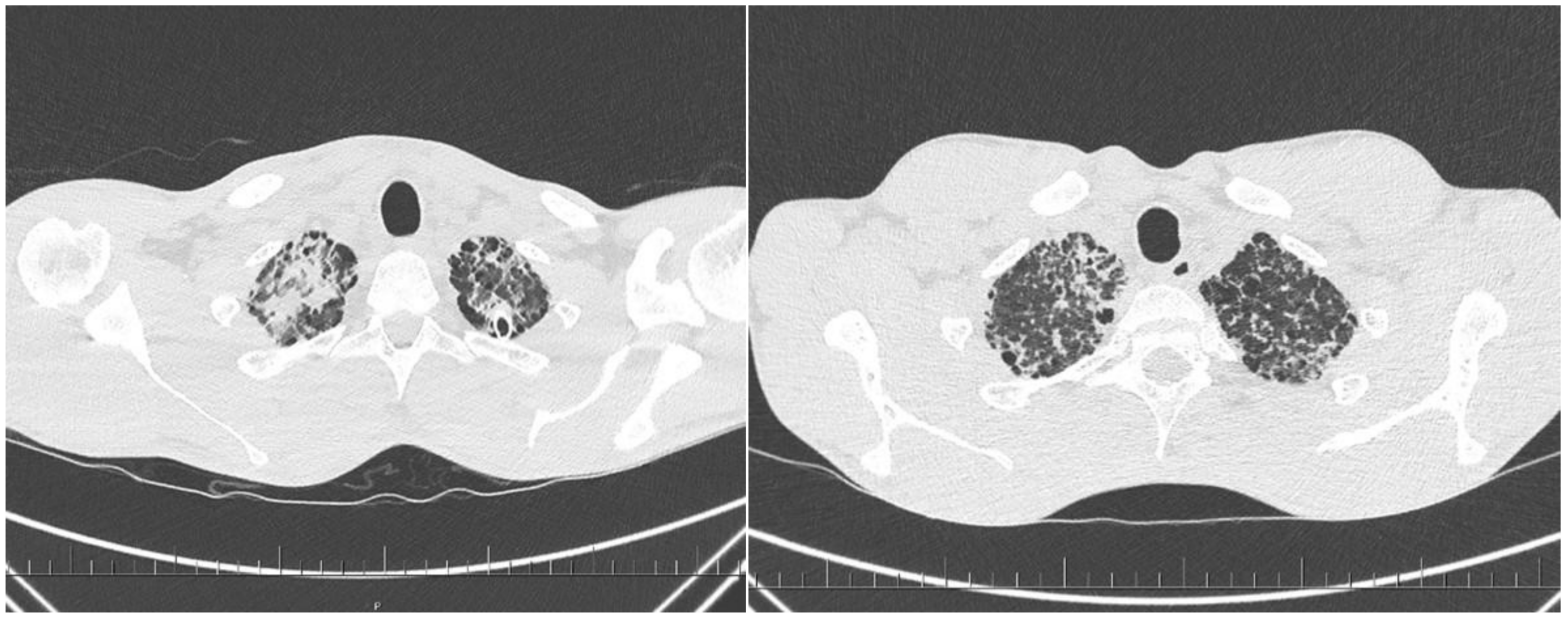
Computed tomography of the lungs revealed a massive interstitial pathological process in superior **(A)** and inferior pulmonary lobes **(B)**. Histology of the left lung confirms the presence of proteinosis.

**Figure 2:**
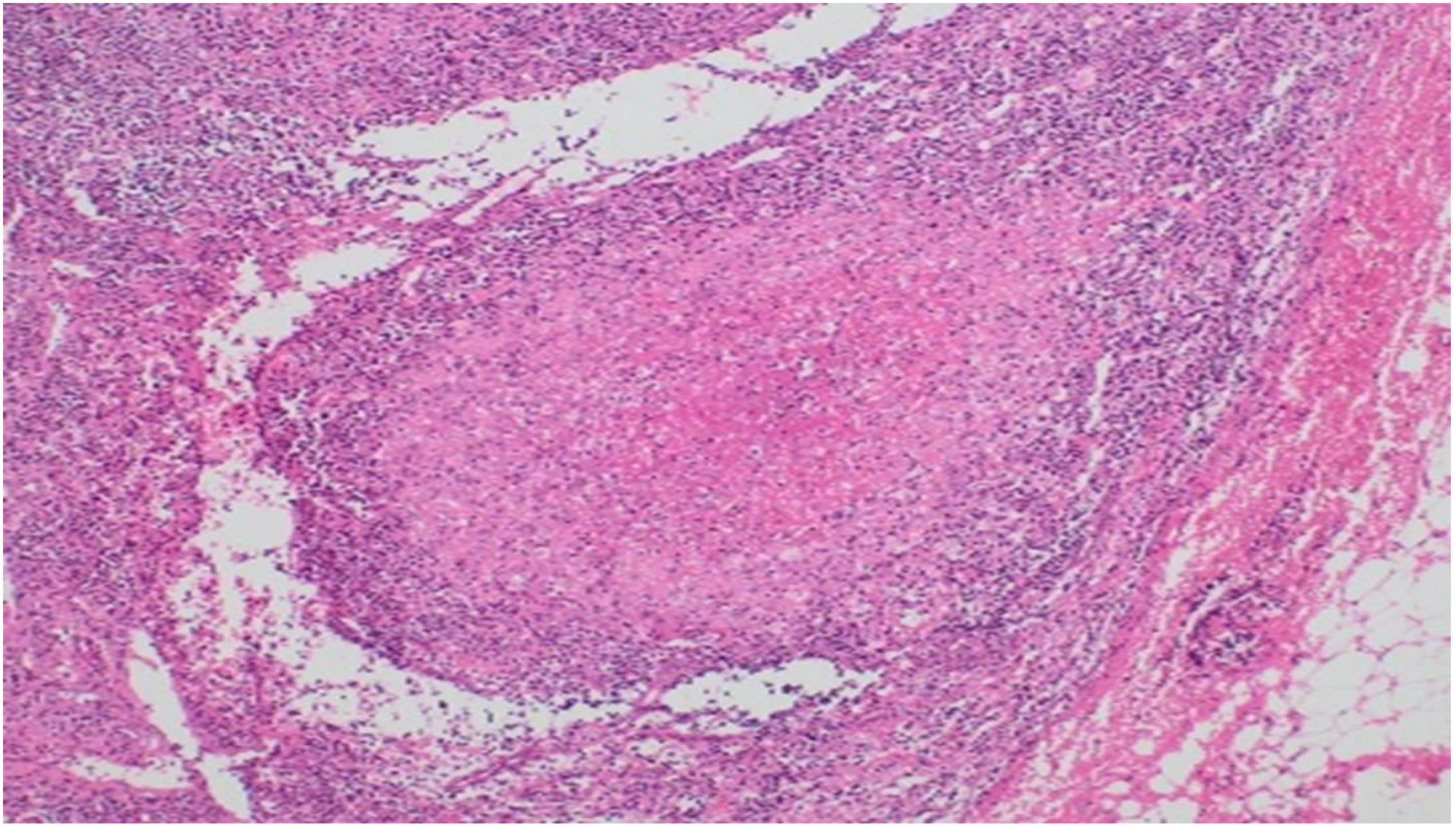
Granuloma with fibrinoid necrosis from cervical lymph node.

This pertains to a Caucasian man born in 1995 with no positive family history of hematopoietic and immune system diseases. Past medical history was positive for recurrent pneumonia, orchitis, and infectious complications caused by bacterial and viral etiologies in teenagers. At the age of 14, he overcame bilateral influenza pneumonia (H1N1) complicated by the development of lung abscesses and bacterial superinfection. Epstein–Barr virus and cytomegalovirus were repeatedly found to be negative by PCR tests, and special tests for mycobacterial infections were negative from bronchoalveolar fluid. A dominant etiology of the infections was Gram-positive bacterial infection. After another course of bronchopneumonia at 23 years of age, a CT scan confirmed interstitial lung disease and splenomegaly (**Figure 1**). The recommended lung tissue biopsy was refused. At the age of 26, he was sent for hematological examination because of pancytopenia, generalized chest, mediastinal, and abdominal lymphadenopathy. Complex hematological examinations were conducted, considering bone marrow aplasia, myelodysplastic syndrome, paroxysmal nocturnal hemoglobinuria, and malignant lymphoma. The blood count revealed mild anemia, grade 2 thrombocytopenia, lymphopenia grade 1, and monocytopenia. A review of previous medical pathology verified the presence of monocytopenia at a younger age; it was first mentioned at 14 years old. Bone marrow examination demonstrated mild hypocellularity of hematopoiesis and dysplastic changes in the erythrocytes. However, this did not fulfil the criteria for MDS. Bone marrow morphology and flow cytometry revealed a decrease in monocyte and B lymphocyte counts compared to the normal range. Cervical lymphatic node histology has been described as difficult-to-classify necrotising (lympho) histiocytic (granulomatous) proliferation, suggesting a primary consideration of systemic immunopathological disease, with infectious disease being less likely. No convincing signs of malignancy were observed (**Figure 2**). Shortly after the initial evaluation, the patient experienced an episode of immune thrombocytopenia with prompt response to corticosteroids.

**Figure 3:**
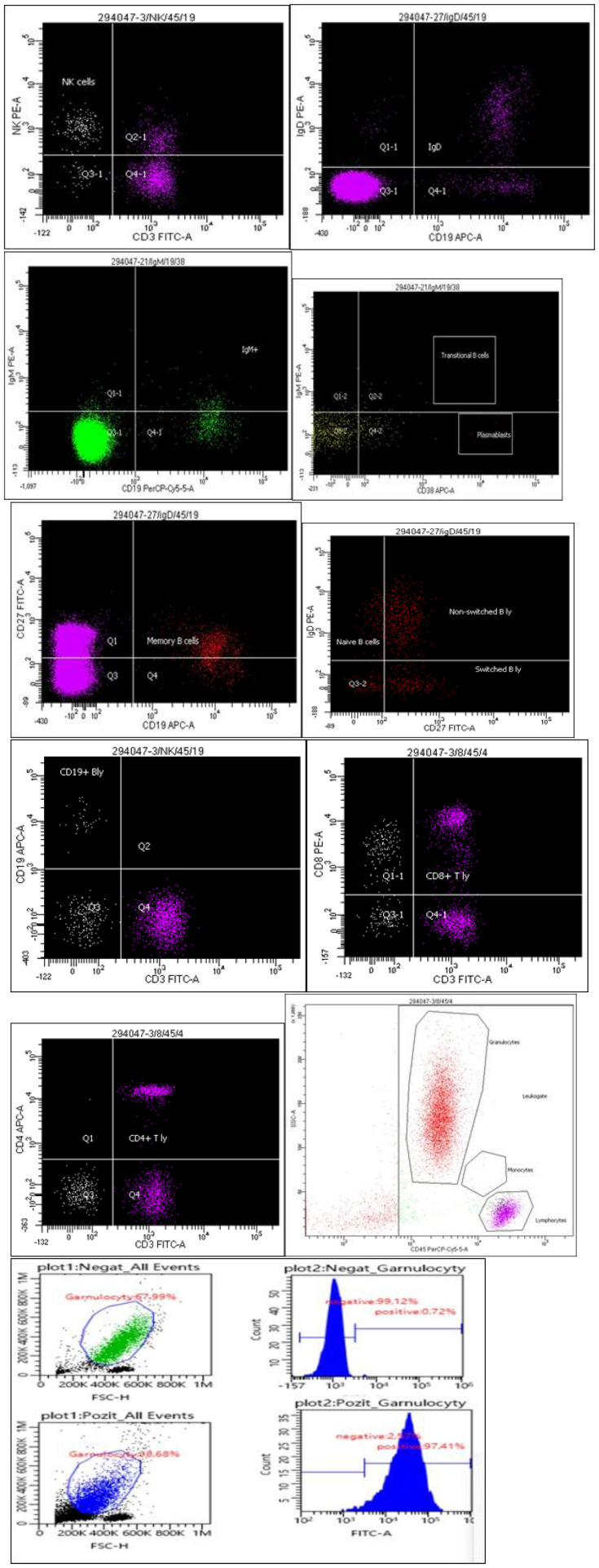
Flow cytometry of peripheral blood. Detected, 25% of lymphocytes; approximately 71% were granulocytes. The monocyte population was not detected **(A)**. **(B, C)** T lymphocyte population represents approximately 88% and shows a reduced CD4/CD8 ratio (0.91), but without significant one’s abnormalities in the expression of analyzed T lymphocyte markers. T lymphocyte activation was not observed. **(D)** Marked B lymphocytopenia (represents 2% of lymphocytes) that was observed in both relative and absolute count. An increased subpopulation of memory non-switched B lymphocytes was detected **(E)**. B lymphocytes predominantly with the phenotype of memory B cells, consisting approximately 70% of the total B lymphocytes **(F)**. A population of transient B lymphocytes was not detected **(G)**. Observed reduced expression of surface IgD as well as IgM on B lymphocytes **(H, I)**. NK cells without pathology **(J)**. Phagocytic activity of neutrophil granulocyte showing reduced respiratory values’ flare-ups (SI-28) **(K)**.

At the age of 27, a histological examination of the lungs revealed proteinosis of the lung, an ambiguous interstitial process, most likely an alveolar proteinosis. During this hospitalization, severe infectious complication developed with severe sepsis and SIRS (systemic inflammatory response syndrome), based on a peritonsillar abscess. A broad-spectrum antibiotic was administered, which demonstrated a good response. At this stage of disease progression, investigation into an underlying primary immunodeficiency was warranted. In humoral immunity, the levels of IgG, IgM, and IgA were normal, with elevation of IgG1 and decrease in IgG2 and anti-pneumococcal IgG. Cellular immunity verified the absence of a monocyte population. A decrease in B lymphocytes was observed with an elevation of memory non–switch B lymphocytes. Transient B lymphocytes were not detected, and a low expression of surface IgD and IgM was observed (**Figure 3**).

Genetic analysis of PID (primary immunodeficiency) panel was performed in the Laboratory of Genomic Medicine, GHC GENETICS SK in Bratislava. DNA was isolated from peripheral blood using a commercial kit, and further examination was established on the clinical exome sequencing platform focused on a panel of 477 genes associated with immunopathies and immunodeficiencies. Variant c.694_695insA p. (Gly232Glufs*50) (reference sequence NM_032638) was identified in the patient in the GATA2 gene in the heterozygous state (49,5%, depth 190), which was classified as probably pathogenic (according to ACMG criteria PVS1 very strong, PM2 supporting) according to Varsome and as probably pathogenic/pathogenic variant (4/5) according to the Franklin database (according to ACMG criteria PVS1 very strong, PM2 moderate). The DNA variant was very rare, identified in only one sample from all of our analyses and one sample from the community (145,000 samples). The variant was not published in the accessible literature, had no rs ID, and was not listed in any population databases. It was located in exon 3 of 6, within three of the four protein domains located downstream of the mutated site. The variant is predicted to cause nonsense mediated decay (NMD), resulting in no functional protein produced from this allele. Bone marrow karyotype presented normal chromosomal constitution 46, XY. The patient was referred to the Slovak Centre for allogeneic hematopoietic stem cell transplantation but did not agree with the procedure. The relatives—parents and brother—did not agree to genetic tests. No other patients with this finding were detected. The patient is on supportive treatment with granulopoiesis growth factors (filgrastim) when the absolute neutrophil count drops below 1.5×10^9^/L, and antibiotic therapy is initiated at the early signs of a bacterial infection. In case of infections, viral, fungal, mycobacterial, and atypical mycobacterial etiology is differentiated. This therapy is given until now, and the patient can work and perform activities of daily living.

## Discussion

GATA2 haploinsufficiency is a major contributor to MDS/AML in adolescents and young adults. The risk of developing MDS/AML is 6% at 10 years, 39% at 20 years, and 81% at 40 years, respectively, as described in a Belgian–French study (2). GATA2 deficiency is a unique primary immune deficiency, also known as immunodeficiency 21, DCML, or monoMAC (OMIM #614172). Immune defects may appear in adulthood, as the number of hematopoietic stem and progenitor cells (HSPCs) decrease with age. The spectrum of immune cell alterations includes dendritic cells, monocytopenia, loss of transitional B cells, absence of CD56 bright NK cells, reversed CD4:CD8 ratio, excess CD45RA+ CD8+ T cells, and poor humoral response despite normal immunoglobulin levels (3, 4). Donadieu described severe bacterial infections as the most frequent pathogenic occurrences (2). Spinner et al. reported that severe viral infections were the most common ones in their series (70%), especially human papillomavirus (HPV) infections (5). Infections with other disseminated pathogens are frequently observed in GATA2-deficient patients, including non-tuberculous mycobacteria, herpes viruses (varicella zoster virus, Epstein–Barr virus, and cytomegalovirus), and invasive fungal and yeast infections. One of the latest nationwide study, an Italian cohort of 30 patients with GATA2 deficiency, revealed new clinical phenotypes—pilonidal cyst/sacrococcygeal fistula, cholangiocarcinoma, and gastric adenocarcinoma—and show that lymphedema may be associated with null/regulatory GATA2 mutation. The median age at the time of first manifestation was 12.5 years, and 55% of patients underwent allogeneic HSCT (3, 5, 9, 12).

The patients can also present with autoimmune manifestations, described in 11%–30% of cases, including early-onset osteoarthritis, piezogenic pedal papules, ankylosing spondylitis, and seronegative erosive rheumatoid arthritis (6). Our report describes a case of autoimmune thrombocytopenia. Granulomatous disease in the lymphatic nodes, as in our case, has rarely been described. Few cases have described ocular and pulmonary involvement. Other cases have reported granulomatosis in the pleura, pericardium, skin and muscles, and lymph nodes (7).

Along with monocytopenia in our case, granulomatous disease and pulmonary proteinosis are the dominant symptoms. Pulmonary dysfunction is a common finding in up to 50% of patients with GATA2 deficiency, even in the absence of a hematopoietic disease. In addition to recurrent infections, pulmonary alveolar proteinosis (PAP) is one of the most distinctive features of the lungs (8). Monocytopenia is a critical biological clue for a correct diagnosis. Chronic monocytopenia should never be neglected as there are few diagnostic possibilities in adults, including myelosuppression, hairy cell leukemia, and GATA2 deficiency.

Allogenic transplantation of hematopoietic stem cells is currently the only curative method for GATA2-associated MDS/AML and immune dysfunction. Transplantation can be especially challenging in this disease due to concurrent infections, pre-existing leukemia, or transformation of myelodysplastic syndrome into leukemia. A recently published paper presented the long-term results of 67 adult patients with GATA2 deficiency after allogenic hematopoietic stem cell transplantation. The overall survival (OS) at 1 and 5 years was 83% and 72% (median follow-up, 5.6 years), respectively. The factors associated with worse OS were the year of HSCT, a history of excess blasts before transplant, and peripheral blood stem cell (PBSC) grafts. Age at HSCT, non-myeloablative conditioning, and PBSC grafts were associated with increased non-relapse mortality. The outcome of PAP patients after HSCT requires larger studies to evaluate the real benefit and risk in this indication. The rationale for efficacy is via the reconstitution of the alveolar macrophages’ function. In a recent study, only five patients with PAP were transplanted. PAP disappeared in two of the five affected patients; the three other affected patients died, including two from respiratory distress. While the direct link between PAP and cause of death could not be established, this raised concerns about the risk of pulmonary complications in those patients. This is in line with the severe outcome observed by Marciano et al. (11), who reported a crude mortality rate of 54% in patients with PAP. The duration of PAP before HSCT and the appearance of fibrosing lesions may explain the negative impact in some patients. Bone marrow monitoring to identify clonal evolution and perform HSCT before the appearance of excess blast is mandatory (10).

The patient signed an informed consent and agreed with the publication of the case report. There is no conflict of interest in processing this manuscript.
