# Correction: GATA2 deficiency in an adult with alveolar proteinosis, infections, lymphadenopathy with granulomatosis, and immune deficiency: case report

**DOI:** 10.3389/fimmu.2025.1750971

**Published:** 2025-12-11

**Authors:** Juliana Gabzdilová, Štefan Raffáč, Gabriela Krasňanská, Michal Konečný

**Affiliations:** 1 Department of Haematology and Oncohaematology, P.J.Šafárik Medical Faculty and L.Pasteur University Hospital, Kosice, Slovakia; 2 Department of Clinical Immunology and Allergology, L.Pasteur University Hospital and OKIA s.r.o, Košice, Slovakia; 3 Laboratory of Genomic Medicine, GHC GENETICS SK, Bratislava, Slovakia; 4 Faculty of Natural Sciences, Department of Biotechnology a Biology, University of Ss. Cyril and Methodius in Trnava, Trnava, Slovakia

**Keywords:** monocytopenia, immunodeficiency, infections, alveolar proteinosis, granulomatosis of lymphatic tissue, autoimmunity

The order of authors in the author list of the published paper was erroneously given as:

“Štefan Raffáč^1*^, Juliana Gabzdilová^2^, Gabriela Krasňanská^3,4^, Michal Konečný^3,4^”

The correct author list reads:

“Juliana Gabzdilová^2^, Štefan Raffáč^1*^, Gabriela Krasňanská^3,4^, Michal Konečný^3,4^”

The original version of this article has been updated.

